# Modified cognitive behavioral therapy approach reduces loudness discomfort levels for an autistic child with hyperacusis: case report

**DOI:** 10.3389/fpsyt.2024.1440624

**Published:** 2024-10-30

**Authors:** Tana B. Carson, Lisa A. Guerrero, Monica Niebles, Cindi G. F. Gayle

**Affiliations:** ^1^ Department of Occupational Therapy, University of Florida, Gainesville, FL, United States; ^2^ UF Health Rehab Center for Kids, Shands Hospital, Gainesville, FL, United States; ^3^ Department of Occupational Therapy, Florida International University, Miami, FL, United States; ^4^ Department of Special Education, School Psychology, and Early Childhood Studies, University of Florida, Gainesville, FL, United States; ^5^ Little Leaves Behavioral Services, Weston, FL, United States; ^6^ The Florida Obsessive Compulsive Disorder (OCD) Autism and Anxiety Treatment Center (FLOAAT Center), Gainesville, FL, United States; ^7^ Department of Psychiatry, University of Florida, Gainesville, FL, United States

**Keywords:** autism, hyperacusis, decreased sound tolerance, treatment, case report, auditory, sensory over responsivity

## Abstract

**Introduction:**

Hyperacusis is common among the autistic population, with a lifetime prevalence estimated at up to 60% compared to 17.1% in those without autism. For autistic children, avoidance behaviors and distress associated with hyperacusis significantly disrupt participation in everyday routines including academic, social and leisure activities. Although hyperacusis is a significant problem for children with autism and their families, there is little research on effective interventions. This report describes the clinical case of an 11-year-old boy with autism who received a modified cognitive behavioral therapy (CBT) approach to address symptoms of hyperacusis.

**Case presentation:**

Patient A is an 11-year-old boy with autism and hyperacusis. He and his parents report difficulties tolerating loud or high-pitched sounds including whistling, fireworks, traffic and high-pitched musical instruments (e.g., the ocarina and flute). When hearing these sounds during everyday activities (e.g., celebrations and social events) he will often ask strangers to stop, cover his ears, or avoid/run away from the source of sound. A modified CBT approach was combined with exposure therapy, and sensory-based self-regulation strategies to improve tolerance and decrease distress when hearing whistling. Treatment outcomes include improved loudness discomfort levels in audiology evaluations, improved auditory domain scores on the Sensory Profile questionnaires, lower self-reported subjective units of distress scale (SUDS) ratings in response to bothersome sounds, and decreased use of noise canceling headphones during daily activities. The client and his parents also reported generalization of these improvements with other sounds (e.g., fireworks).

**Conclusion:**

The patient described in this case report showed measurable improvements in his ability to tolerate whistling, a bothersome sound encountered regularly in his daily life. Considering the high prevalence rate of hyperacusis in autism and its impact on children and family routines, stress and daily living, the development and testing of an effective treatment approach for hyperacusis is needed. The treatment plan for this case arose from the collaboration between professionals in occupational therapy, applied behavior analysis, audiology, and clinical psychology. Future studies are encouraged to determine the efficacy of this combined approach for other children with autism and hyperacusis.

## Introduction

Hyperacusis symptoms are more commonly reported among autistic individuals (60.1%) (1, 2) than non-autistic individuals (up to 17.1%) (3). Hyperacusis involves a painful reaction to the physical characteristics of sound often eliciting physical reactions to escape or block the bothersome sound (4). In addition to avoidance, individuals with hyperacusis also report symptoms of emotional distress including stress, anger, irritation, anxiety, and depression (5). For autistic individuals, emotional reactivity and avoidance of sounds can significantly disrupt socialization and participation in activities within natural environments (6–8). For children, the long-term implications of these aversive responses to sensory stimuli may later impede functioning as an independent adult (4).

There are several self-regulation interventions available with varying levels of evidence. Applied relaxation (9) and mindfulness-based techniques (10, 11) are commonly reported to be effective for adults and evidence in pediatric populations is emerging (12). Sensory-based self-regulation programs involving child-friendly educational strategies (e.g., visual aids illustrating arousal level as analogous to car engines/thermometers) and sensory modulation techniques (e.g., calming deep proprioceptive input) have gained popularity among clinicians working with pediatric populations (e.g., The ALERT Program^®^ and Zones of Regulation). Evidence of effectiveness for these techniques is limited, but promising (13, 14). Self-regulation differences are common in autism (15–17) and should be considered when developing treatment plans for hyperacusis related distress in this population.

Hyperacusis treatment strategies available to rehabilitation professionals (e.g., occupational or speech therapy) are primarily limited to over-protection (e.g., noise-canceling headphones/earmuffs) and auditory integration listening systems. Although noise-canceling headphones are commonly used, they are problematic as a long-term solution for several reasons: (1) noise canceling or dampening can lead to further hyper-sensitivity/reactivity, (2) they are socially conspicuous and may impair social participation/lead to bullying, etc. (18), and (3) they may negatively reinforce avoidance of/escape from auditory stimuli. Auditory integration training (AIT) involves listening to electronically altered auditory stimuli through headphones. Although individual, anecdotal benefits may be reported, randomized controlled trials have found this intervention largely ineffective (19, 20). Considering the high prevalence of hyperacusis and its impact on patients and their families, it is necessary to establish effective strategies for addressing this condition in autistic children.

Cognitive behavioral therapy (CBT) with exposure therapy (ET) can be effective for adults with hyperacusis (21–23, 45). Historically, autistic children respond well to CBT with and without ET (24–30). Thus, it is reasonable to suspect that this approach could also be useful for reducing anxiety, distress, and avoidance associated with hyperacusis in autistic children. However, few studies have explored the effectiveness of CBT with ET for hyperacusis in autism specifically (31, 32).

Although difficulty tolerating sounds is a commonly reported problem for autistic patients and their families, there is currently no “gold standard” evidence-based treatment available for those with autism. This report presents a clinical case of hyperacusis in an autistic child that was addressed successfully using CBT with an ET approach modified as recommended for autistic individuals (33, 34) and combined with sensory-based self-regulation strategies.

## Patient information

Patient A is an 11-year-old boy with a previous diagnosis of Pervasive Developmental Disorders-Not Otherwise Specified (4th ed., text rev.; *DSM–IV–TR*; 35) and complaints of difficulty tolerating loud or high-pitched sounds (e.g., whistling, fireworks, car traffic and high-pitched musical instruments. He and his parents report avoidance behaviors in response to these stimuli including asking strangers to stop whistling, covering his ears, escaping/running away and fearful avoidance of certain events/activities/people where whistling may occur (e.g., celebrations and events).

## Clinical findings and assessments

Parent and patient questionnaires and clinical interviews were collected before, during and after the intervention to determine the effect of treatment on outcomes of interest including loudness discomfort levels (LDLs), behavioral responses to sounds (Sensory Profile), and distress (SUDS).

### Audiological evaluation and loudness discomfort levels (LDLs)

A comprehensive audiological test battery was completed pre- and post- treatment by an audiologist. Tympanometry, distortion product otoacoustic emissions, speech audiometry in quiet, and pure tone audiometry via air and bone conduction were conducted. Loudness discomfort levels (LDLs) were assessed for speech and pure tones at various presentation sound levels expressed in decibels Hearing Level (dB HL), and at various frequencies (pitches), expressed in Hertz (Hz).

### Pre-intervention LDLs

**Figure 1:**
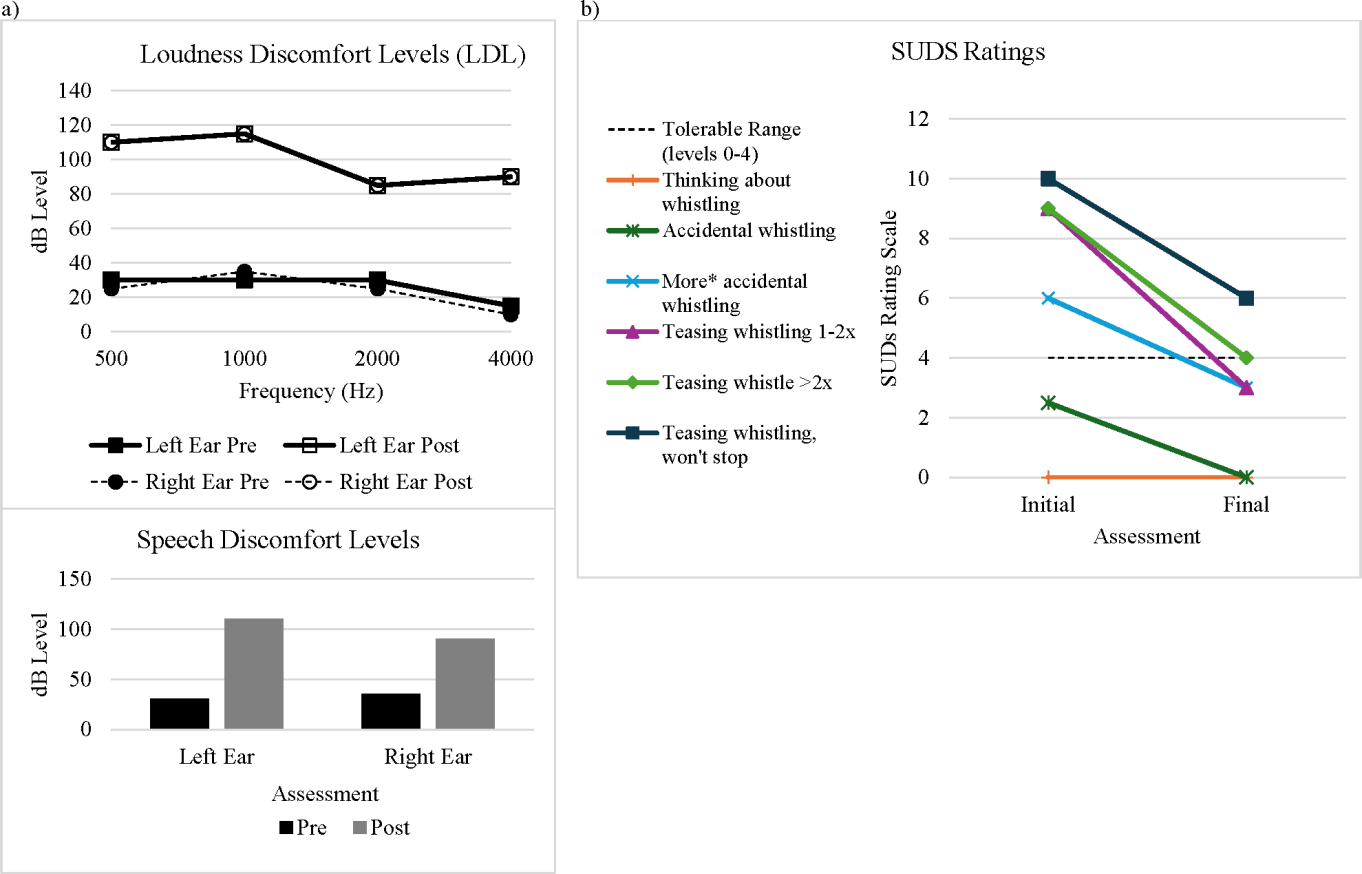
Treatment outcomes. **(A)** Loudness discomfort levels (LDL) and speech discomfort levels in right and left ears taken before (pre) and after (post) treatment in audiology and **(B)** subjective units of distress pre- and post-treatment for whistling stimuli. *“More” indicates higher pitch, higher sound level, or longer duration.

The audiology report pre-intervention confirmed that the patient demonstrated normal hearing bilaterally. The patient provided reliably repeatable LDLs which were consistent with hyperacusis (i.e., LDLs below 90 dB HL for pure tone frequencies) (36). Patient A’s initial discomfort response level of 60 dB HL for a 1000 Hz pure tone prompted re-evaluation with a starting level of 0 dB HL. He reliably reported discomfort at 25-35 dB HL for 500-2000 Hz tones and speech, and 10-15 dB HL for 4000 Hz pure tones (**Figure 1A**). Levels did not change significantly despite repeated re-instruction and were a reliably repeatable response; however, these results are not considered a valid measure of loudness discomfort per se, as the patient was able to tolerate average conversational speech without apparent distress at a level greater than 50 dB HL outside the testing booth. Furthermore, results for 4000 Hz revealed apparent thresholds of loudness discomfort at sound levels below those occurring in unoccupied rooms with ambient noises like air conditioners, whereas he was able to tolerate sitting in such a “quiet” room outside the testing booth without any reported discomfort. Hyperacusis was diagnosed without classification of severity, based primarily on patient self-report of having an aversion to any nonspecific sound as a function of intensity and based on parent report of his atypical behaviors in response to sound. Although parts of his LDL audiological assessment were considered invalid, these measurements may still serve as a useful baseline by which to evaluate the effects of treatment.

### Sensory profiles

The Sensory Profile Caregiver Questionnaire (37) and Sensory Profile-2 Child (38) are standardized parent-report questionnaires that evaluate response patterns to sensory stimuli. Sensory Profiles were collected at pre-intervention, mid-intervention and post-intervention. For this study’s purpose only the auditory domain is reported. Raw data for additional categories of the Sensory Profiles are reported in the **Supplementary Table 1**. At pre-intervention the Sensory Profile Caregiver Questionnaire auditory domain score was 23 “definite difference”.

### Subjective units of distress scale (SUDS)

The SUDS (39) is a subjective measurement tool used to assess patient pain, anxiety, or discomfort on a 10-point scale. A sound distress hierarchy was developed in collaboration with the patient and his parent to describe and rank whistling stimuli by SUDS levels(**Table 1**). The sound distress hierarchy was used as a baseline measure, as a guide for ET and post-intervention measure. SUDS ratings were also used throughout treatment to monitor responses to each exposure activity.

**Table 1:** Whistling sound distress hierarchy for patient A.

SUDS	Self-reported Response to Sounds at Initial Assessment
0	thinking/talking about whistling
1	hearing whistling on accident or just 1x then stops; short, low-pitched whistle
2	hearing whistling on accident or just 1x then stops; longer, higher-pitched whistle than level 1
3	hearing whistling on accident or just 1x then stops; longer, higher-pitched whistle than level 2
4	hearing whistling on accident or just 1x then stops; longer, higher-pitched whistle than level 3. *Note: This is the highest level considered to be tolerable to patient at intake.*
5	hearing whistling 2x or more; if someone else is whistling (i.e., not himself), he must cover his ears and ask someone to stop
6	hearing whistling 2x or more but louder/higher pitched than level 5; if someone else is whistling (i.e., not himself), he has to cover his ears and ask someone to stop
7	hearing whistling 2x or more but louder/higher pitched than level 6; if someone else is whistling (i.e., not himself), he has to cover his ears and ask someone to stop
8	hearing whistling done on purpose 1-2x to bother me (e.g., whistling that continues after he has asked them to stop, example: teasing from sibling)
9	hearing whistling done on purpose 1-2x but louder/higher pitched than level 8 to bother me (e.g., whistling that continues after he has asked them to stop, example: teasing from sibling)
10	hearing whistling done on purpose/more loudly/without stopping even when I’m yelling for them to stop*

*Patient A reports that there is a level 100 that he describes as “off the charts” when hearing whistling done on purpose/more loudly/without stopping by 1 or more people (e.g., sibling and his friends) to bother me.

## Therapeutic intervention

Patient A received a modified CBT and ET in an outpatient clinical setting for a total of 34 30- to 45-minute sessions at 1x/week. The intervention included two steps- (1) psychoeducation and (2) auditory ET. Patient and caregiver education and home program development occurred throughout all sessions. Treatment also modified according to the National Institute for Health & Care Excellence (NICE) guidelines for improving the effectiveness of CBT for autistic populations (34) including (a) emotional recognition training, (b) the use of visual aids, (c) rest breaks, (d) a structured and concrete approach, (e) involving parents and caregivers in the intervention planning and (f) implementation, and incorporating the child’s special interests into therapy. Additional modifications based on clinical reasoning included: (1) progressing through the sound distress hierarchy at a slow pace, (2) incorporating client-directed play-based activities (e.g., whistling in Morse code to guess letters and solve a puzzle) and (3) applying sensory-based self-regulation strategies as needed to allow the patient to remain engaged in ET without undue anxiety/distress.

### Psychoeducation

#### Interview, parent report, and self-report

During the first 6 sessions, psychoeducation focused on: (1) assessing patient and caregiver insight into the problem, (2) identifying the impact avoidance behaviors have on the patient and family (3) identifying patient and caregiver goals for treatment and (4) establishing a sound distress hierarchy. During the psychoeducation sessions the patient and caregiver demonstrated improved insight into the problem of hyperacusis and the impact of symptoms on daily life and identified whistling as the primary stimulus of concern (**Table 2**).

**Table 2:** Patient intake interview.

Question:	Answer:
What sounds bother you?	Whistling, fireworks, loud/high-pitched sounds, traffic
Which of these sounds bothers you the most?	“all of them”
Which sound would you like to work on?	Whistling
What do you do when you hear this sound (whistling)?	Cover ears, ask politely for the person to stop whistling (even if they are a stranger), hide/escape to quiet place, avoid certain situations (e.g., school assembly, awards ceremonies, shows).
Do you think it is possible to hear this sound with less distress/discomfort?	“No. It physically hurts. Hearing my [sibling’s] ‘atomic whistle’ is like having fireworks strapped to my ears.”

#### Emotional self-regulation

The Alert Program^®^ was utilized to improve the patient’s ability to self-regulate before, during, and after auditory challenges (40). The focus of the self-regulation treatment was for the patient to be able to: (1) consistently and accurately identify his emotional/arousal level as too high, too low, or just right, (2) identify potential strategies to modify if too high/low and (3) effectively utilize regulatory strategies in real-time. Strategies from Social Thinking (41) were also included to improve insight regarding avoidance behaviors and impacts on social engagement.

### Exposure activities and treatment timeline

A sound distress hierarchy served as a baseline measure and a guide for developing exposure activities (**Table 1**). The patient agreed to start at the lowest level (0) with imaginal exposures such as thinking about and talking about whistling. Once he could engage in an exposure activity with a self-reported SUDS level 4 or below, he progressed to the next level in his hierarchy. Each activity was developed in collaboration with the patient and endeavored to incorporate his interests and an element of fun. For example, his first exposure was virtual and involved alternating funny cat videos and World Championship of Whistling videos on YouTube. The second exposure was *in vivo*, in real life, and involved the therapist whistling at a low level and for a short time (less than 1 second) and at expected times only. Later *in-vivo* exposures progressed through the hierarchy with incremental increases in *loudness* (high being more difficult than low), context (who is whistling- self being easier than others), *quality* (poor quality whistling more difficult than good quality), *distance* from the sound source (near being more intense than far), *duration/frequency* (long/repetitive whistling being more bothersome than a quick/singular whistling) and *timing* (expected/unexpected). ET did not actively prevent the participant from engaging in avoidance behaviors. The therapist encouraged the use of self-regulation strategies to decrease anxiety/distress and allow the participant to actively engage in exposure activities. Distress was continuously monitored using the SUDS ratings within a mutually agreed-upon range (i.e., 4/5 or less) throughout the treatment timeline (**Table 3**). Upon mastery of *in vivo* exposures in session, home exercise programs (HEP) were developed for the client to practice exposures in other contexts (e.g., home, school, and the community) as appropriate to encourage generalization to other contexts.

**Table 3:** Timeline of CBT with exposure therapy sessions.

	CBT Session	Brief Description of Exposure Activity	SUDS Ratings	Notes
Psychoeducation and Imaginal Exposures	1	Thinking or talking about whistling	N/A	Psychoeducation activites including: motivational interviewing, developing sound distress hierarchy, exploring self-regulation strategies, myth busting activities (e.g., reviewing audiology results and evidence that the patient demonstrates normal (undamaged) hearing)
	2	Thinking or talking about whistling	N/A	
	3	Thinking or talking about whistling	N/A	
	4	Thinking or talking about whistling	N/A	
	5	Thinking or talking about whistling	N/A	
In vivo Exposures in Session	6	videos of whistling at low sound level; short duration (<10 sec)	5	Exposure activities with incremental increases in sound intensity (based on slight changes in duration, frequency, sound level, and context) within the agreed range of SUDS (5 or below)
	7	videos of whistling at low sound level; short duration (10-20 sec)	5	
	8	videos of whistling at medium sound level, short duration (<10 sec)	5	
	9	OT whistling “happy birthday song” with OT standing in hallway and patient in treatment room x1 trial	3	
	10	mom whistling in room for 5 seconds x3 repetitions	0	
	11	patient whistling in room for 5 seconds x3 repetitions	3,3,3	
In vivo Exposures at Home and/or in Session	12	OT whistling during morse code activity to x6 morse code letters	2	Home exercise program (HEP) developed and updated each session in addition to continuing to progress through hierarchy with therapeutic activities and exposures in session.
	13	Mom whistling 5’ from patient	2	
	14	Patient whistling for 3 seconds	5	
	15	Rehab aide whistling in hallway 2 seconds	3	
	16	OT whistling 5 feet away for 2 seconds	8*	
	17	Marco Pollo with OT and pt whistling loudly x 15 min	1	
	N/A	HEP reviewed for Ocarina	N/A	
	18	Marco Pollo with OT and patient whistling loudly throughout the game for 15 minutes total	0	
	19	Sound Distress Hierarchy reviewed	“nothing over 5” 2 months	
	20	HEP reviewed/revised; patient reports feeling discomfort when he feels “surprised” by the sound	N/A	
	N/A	Reviewed pain scale; introduced improved pain scale; completed OT reassessment	N/A	
	21	Dad whistling during obstacle course x 30 whistles	4	
	22	OT whistling- 1: long, low pitched whistle (5 sec), 2: long, low pitched whistle (7 sec), 3: long, low pitched whistle (8 sec)	6**, 2, 2	
	23	Self-whistling- 1: short, high pitched (3 sec); 2: long, high pitched (7 sec); 3: short, high pitched (2 sec)	3, 3, 3,	
	24	OT whistling- medium, high-pitched whistle unexpectedly (1 sec) intermittently throughout	4	
	25	OT intern whistling- short, low whistles x 4	0	
	26	self-whistling x4	4	
	27	OT intern blowing plastic whistle x 1	6, 3	
	28	OT long, high pitched whistle x 4 (3-5 sec each)	0, 1, 0, 0	
	29	OT intern blowing plastic whistle short x 3, medium x 1, long x 1	0, 0, 0, 6.5***	
	30	Walking outside of building with OT whistling (expected) x 6 trials	4	
	31	Walking outside of building with OT whistling randomly (unexpected) x 6 trials	2	
	32	New public spaces x2 with OT whistling x2 in each space	2	
	33	Sibling “atomic whistle” with sibling seated in car and patient outside of car x 12 trials (3-5 sec/each) with pt controlling start/stop of whistle	2, 3, 7****	
	34	Sibling low whistle with pt seated in car with sibling	3, 4	
	N/A	OT Goal Progress assessed; HEP reviewed/updated	N/A	
	N/A	Sound Distress Hierarchy re-assessed; HEP reviewed/updated		

N/A = denotes sessions that the patient was also working towards other OT goals (e.g., coordination) and hyperacusis was not the primary focus.

*No visible signs of discomfort. FACES pain scale level 2 observed during whistling and 0 afterwards with patient smiling/laughing. **OT immediately backed down the intensity of activities after patient reported level 6 with visible signs of discomfort. ***Patient put his head down but was smiling and able to continue talking immediately following long whistle (FACES level 2 observed during whistle). ****Patient reported level 7 on final trial, however, he was observed to be laughing, talking, and walking while reporting level 7, which is not consistent with the improved pain scale that the patient and therapist agreed to reference for SUDS (e.g., level 7 is described by crying or being unable to function). Patient’s parent and OT agreed that the patient was demonstrating responses more consistent with his previous level 3-4 responses rather than 7.

## Outcomes

Patient A completed 34 sessions of CBT with ET focusing on improving tolerance for whistling sounds.

### Post-intervention LDLs

LDLs were re-evaluated for Patient A following treatment using the same language used at his initial evaluation. For consistency, stimuli were initially presented at 0 dB HL and raised in 5 dB increments. Post-intervention he did not report loudness discomfort for speech until 90 dB HL in the right ear and 110 dB HL in the left ear (**Figure 1A**). Notably, he stated that he was not responding to discomfort, but a tickling sensation in his ear canals. In sharp contrast to his visible distress during initial evaluation, he was laughing throughout this portion of testing. He was able to tolerate the maximum presentation levels available on testing equipment for 500 and 1000 Hz pure tones with no report of discomfort. However, he did become visibly anxious when 2000 and 4000 Hz pure tones were tested and reported loudness discomfort at 85 dB HL bilaterally. Although several tests improved to within normal limits (500 Hz, 1,000Hz and speech), some remained below normal limits (2,000Hz and 4,000Hz) indicating that Patient A still meets criteria for hyperacusis. Anecdotally, the client reported only one instance of wearing noise-canceling headphones in the past month while attending one of several fireworks displays, whereas he was previously using hearing protection in everyday scenarios.

### Sensory profiles

The Sensory Profile questionnaires provide categorical outcome variables comparing the patients’ responses to age-matched peers. At mid-intervention the auditory domain of the Sensory Profile Caregiver Questionnaire improved to a score of 27 “probable difference” and at post-intervention the auditory domain of the Sensory Profile-2 Child to a score of 24 “just like the majority of others”. Other areas of the Sensory Profile are reported in the **Supplementary Table 1**.

### Emotional self-regulation

Throughout treatment, Patient A demonstrated improvements in self-regulation before, during, and after auditory exposure activities. At post-intervention he could consistently and accurately identify his arousal level as too high, too low, or just right on more than 75% of opportunities. He could also effectively modulate his arousal level in real-time when feeling distressed with minimal to no cues from adults on over 75% of opportunities presented in session. According to parent and self-report, he demonstrated decreased avoidance and unexpected behaviors (e.g., covering his ears, asking strangers to stop whistling or escape behaviors) in response to whistling stimuli in everyday situations.

### Exposure challenges and subjective units of distress

Initially, Patient A could not talk about whistling for more than 2-3 minutes without displaying avoidance behaviors like changing the conversation topic or shutting down. After the first two sessions of psychoeducation and imaginal exposures, he demonstrated an improved ability to discuss whistling, identify different types and rate his levels of distress. By visit six, he could talk about whistling and watch videos of “The Whistling World Championship” for 1-3 minutes continuously (with sound level lowered). He initially reported that SUDS levels 8-10 included an element of “betrayal” from others, resulting in an emotional response in addition to discomfort. Most notably, towards the end of treatment he demonstrated his ability to tolerate his sibling “atomic whistle” (previously described as being incredibly loud and incredibly painful at a level 8-10) at a SUDS level 4 or better on 80% of opportunities (**Figure 1B**).

## Discussion

The patient demonstrated measurable improvements in his ability to tolerate aversive auditory stimuli (e.g., whistling) following a modified CBT with ET approach in an occupational therapy setting. LDLs improved to within normal limits at low frequencies while high frequencies remain consistent with hyperacusis. Parent and self-report sensory questionnaires reflect improvements in the auditory domain from “definite differences” to responding “just like the majority of others” his age. The patient and parents report improved engagement in daily activities with less over-protection, avoidance and distress. While treatment was focused on whistling, Patient A interestingly also demonstrated improved tolerance for other stimuli (e.g., pure tones, speech, and fireworks displays). These additional benefits suggest that treatment may provide a framework of strategies that can be applied successfully to other aversive auditory stimuli once mastered.

Additionally, Patient A demonstrated improved emotional and self-regulation skills as evidenced by identifying and utilizing self-regulation strategies effectively in real time both during and outside of sessions. Initially loud whistling resulted in a SUDS level above 8 and included feelings of “betrayal” or teasing. Although these stimuli remain difficult to tolerate, his self-reported distress decreased from level 8-10 to level 6 or better further illustrating improvements in self-regulation.

### Therapy modifications

Modifications were made to the CBT and ET approaches according to recommendations by Stiegler and Davis (4) and NICE (34) and based on the clinical experience of the authors. The authors suspect that these modifications helped reduce maladaptive behaviors (e.g., escape/avoidance) and intense emotional reactions to facilitate engagement in ET. Importantly, parents and the patient were included as collaborators in the development of all treatment activities. Patient A also reported experiencing benefits to the play-based approach stating that “the pain of whistling is diluted by fun” suggesting that incorporating play may facilitate counter conditioning.

### Limitations

While this approach was successful for this client, questions remain as to whether long-term changes were maintained for this client. Future studies could determine the active ingredients of treatment, long-term outcomes, the utility of booster sessions, and the optimal duration and frequency of treatment. Although IQ testing was not available for this client, he was considered high-functioning and attended mainstream classes. In its current state this approach would not be feasible with a client who cannot communicate their assent or participate in the planning and implementation of exposures. Parent training focused approaches may be more appropriate in those cases and should be explored. The Sensory Profile results should be interpreted with caution since two different versions were used and other interventions were provided simultaneously targeting other sensory features (e.g., feeding aversion).

## Conclusion

This case report illustrates a reduction in hyperacusis symptoms in an 11-year-old autistic boy after receiving 34 sessions of modified CBT with ET. Initially he could not tolerate talking about whistling or hearing whistling at any level. Improvements were observed in LDLs, sensory questionnaires, self-reported distress levels and use of ear protection. The client and his parents also reported improvements tolerating other auditory stimuli (e.g., pure tones, speech and fireworks). Considering the high prevalence of autistic children who experience difficulty tolerating sounds and the related impacts on anxiety (2, 42, 43) family/parental stress (43, 44), and daily activities (18), it is critical to establish evidence-based hyperacusis interventions for this population. The treatment plan for this case arose from the collaboration between professionals in occupational therapy, applied behavior analysis, audiology, and clinical psychology.

## Data Availability

The data analyzed in this study is subject to the following licenses/restrictions: data is restricted to age, diagnosis, prognosis, and treatment related information. Requests to access these datasets should be directed to tcarson@fiu.edu.
