# Supplementary material for: Modified cognitive behavioral therapy approach reduces loudness discomfort levels for an autistic child with hyperacusis: case report
Source: Front Psychiatry. 2024 Oct 30;15:1440624. doi: 10.3389/fpsyt.2024.1440624 (PMC11557304; doi:10.3389/fpsyt.2024.1440624)
Supplement: Supplementary file 1 [file Table1.docx]

**Online Supplement Table 1.** Sensory Profile Questionnaire Raw Scores

|  | **Pre-Intervention** | | | **Mid-Intervention** | | | **Post-Intervention** | | |  |
| --- | --- | --- | --- | --- | --- | --- | --- | --- | --- | --- |
| Questionnaire | Sensory Profile Caregiver Questionnaire | | | Sensory Profile Caregiver Questionnaire | | | Sensory Profile-2 CHILD | | |  |
| Sensory Section | Raw Score | Classification | Sensory Section | Raw Score | Classification | Sensory Section | Raw Score | Percentile Range | Classification | |
| AUDITORY Processing | 23 | Definite Difference *(Range: 8-25)* | AUDITORY Processing | 27 | *Probable Difference (Range: 26-29)* | AUDITORY Processing | 24 | 12-85 | *Just Like the Majority of Others (Range: 10-24)* | |
| VISUAL Processing | 25 | *Definite Difference (Range: 9-26)* | VISUAL Processing | 33 | *Typical Performance (Range: 32-45)* | VISUAL Processing | 13 | 11-82 | *Just Like the Majority of Others (Range: 9-17)* | |
| TOUCH  Processing | 69 | *Probable Difference (Range: 65-72)* | TOUCH  Processing | 67 | *Probable Difference (Range: 65-72)* | TOUCH Processing | 26 | 88-96 | *More Than Others (Range: 22-28)* | |
| VESTIBULAR Processing* | 46 | *Probable Difference (Range: 45-47)* | VESTIBULAR Processing* | 41 | *Definite Difference (Range: 11-44)* | MOVEMENT Processing* | 22 | 86-96 | *More Than Others (Range: 19-24)* | |
| MULTISENSORY* | 25 | *Probable Difference (Range: 24-26)* | MULTISENSORY* | 28 | *Typical Performance (Range: 27-35)* | BODY POSITION Processing* | 15 | 10-89 | *Just Like the Majority of Others (Range: 5-15)* | |
| ORAL SENSORY Processing | 43 | *Probable Difference (Range: 40-45)* | ORAL SENSORY  Processing | 30 | *Definite Difference (Range 12-39)* | ORAL SENSORY Processing | 29 | 88-95 | *More Than Others (Range: 25-32)* | |

**Note*. These categories differ between Sensory Profile Caregiver Questionnaire and Sensory Profile-2
